# Micro-stratification of malaria risk in Nepal: implications for malaria control and elimination

**DOI:** 10.1186/s41182-019-0148-7

**Published:** 2019-03-27

**Authors:** Komal Raj Rijal, Bipin Adhikari, Nabaraj Adhikari, Shyam Prakash Dumre, Mayur Sharma Banjara, Upendra Thapa Shrestha, Megha Raj Banjara, Nihal Singh, Leonard Ortegea, Bibek Kumar Lal, Garib Das Thakur, Prakash Ghimire

**Affiliations:** 1 Central Department of Microbiology, Tribhuvan University, Kirtipur, Kathmandu, Nepal; 2 Centre for Tropical Medicine and Global Health, Nuffield Department of Medicine, University of Oxford, Oxford, UK; 3 Department of Immunogenetics, Institute of Tropical Medicine (NEKKEN), Nagasaki University, Nagasaki, Japan; 4 World Health and Research Centre, Kathmandu, Nepal; 5 World Health Organization (WHO), Country Office, Kathmandu, Nepal; 6 Global Malaria Program, World Health Organization Headquarters, Geneva, Switzerland; 7 Epidemiology and Disease Control Division (EDCD), Department of Health Services, Ministry of Health and Population, Kathmandu, Nepal

**Keywords:** Malaria, Micro-stratification, Elimination, Intervention, Nepal

## Abstract

**Background:**

A significant reduction in malaria cases over the recent years in Nepal has encouraged the government to adopt a goal of “malaria-free nation by 2025.” Nevertheless, to achieve this goal, it is critical to identify the epidemiological burden of malaria by specific regions and areas for an effective targeted intervention. The main objective of this study was to estimate the risk of malaria at Village Development Committee (VDC) level in Nepal based on disease, vector, parasite, and geography.

**Methods:**

In 2012, the micro-stratification of malaria risk was carried out in 75 districts of Nepal. Instruments such as a questionnaire, case record forms, and guidelines for malaria micro-stratification were developed and pre-tested for necessary adaptations. Village Development Committee (VDC)-wise malaria data were analyzed using exploratory statistics and were stratified by geographical variables that contributed to the risk of malaria. To understand the transmission risk at VDC level, overlay analysis was done using ArcGIS 10. To ensure transparent, reproducible, and comprehensible risk assessment, standard scoring method was selected and utilized for data from 2009 to 2011. Thus identified, three major variables (key determinants) were given weights (wt.) accordingly to stratification of the malaria risk (disease burden, “0.3” wt.; ecology/vector transmission, “0.5” wt.; and vulnerability-population movement, “0.2” wt.). Malaria risk in a VDC was determined based on the overall scores and classified into four categories: no risk, low risk, moderate risk, and high risk.

**Results:**

Analyzing the overall risk based on scoring of the total VDCs (*n* = 3976), 54 (1.36%), 201 (5.06%), 999 (25.13%), and 2718 (68.36%) were identified as high-, moderate-, low-, and no-risk categories for malaria, respectively. Based on the population statistics, 3.62%, 9.79%, 34.52%, and 52.05% of the country’s total population live in high-risk, moderate-risk, low-risk, and no-risk VDCs for malaria, respectively. Our micro-stratification study estimates are 100,000 population at high risk. Regional distribution showed that the majority of the high-risk VDCs were identified in the Far- and Mid-western regions (19 and 18 VDCs) followed by Central and Western regions (10 and 7 VDCs) with no high-risk VDCs in the Eastern region. Similarly, 77, 59, 27, 24, and 14 VDCs of the Central, Mid-western, Western, Eastern, and Far-western regions, respectively, were found under moderate malaria risk. Of the low-risk VDCs, 353, 215, 191, 148, and 92 were respectively from the Central, Eastern, Western, Far-western, and Mid-western regions.

**Conclusions:**

The current micro-stratification study provides insights on malaria risk up to the VDC level. This will help the malaria elimination program to target interventions at the local level thereby ensuring the best utilization of available resources to substantially narrowed-down target areas. With further updates and refinement, the micro-stratification approach can be employed to identify the risk areas up to smaller units within the VDCs (ward and villages).

## Background

Malaria, caused by *Plasmodium* species, remains one of the major global health problems, causing nearly half million deaths per year. Malaria is endemic in 91 countries and territories in tropical and sub-tropical zones, spanning all continents of the world (except Antarctica and Australia), with transmission intensities ranging from very low to extremely high [1].

Between 2010 and 2015, malaria incidence among populations at risk (the rate of new cases) fell by 21% globally. In that same period, malaria mortality rates among populations at risk fell by 29% globally among all age groups and by 35% among children under 5 [2].

In recent years, an increasing number of countries such as Armenia, Maldives, Morocco, Turkmenistan, and the United Arab Emirates with low and moderate transmission areas eliminated malaria from their entire territory [3]. With the renewed multi-sectoral efforts and commitment, Southeast Asia region has some of the most pronounced malaria declines, with 5 countries out of 11 reporting decrease of more than 75% of cases from 2000 to 2012 [4]. These figures are encouraging for countries embarking towards malaria elimination.

Overall malaria trend in Nepal for the last 5 years indicates a decline of both clinical and confirmed malaria cases [5]. The country has exceeded the Millennium Development Goals in 2010 (set for 2015) to reduce malaria morbidity and mortality and is in a unique position to move towards elimination [5]. With the significant reduction in malaria burden, the Nepal malaria program has set up the vision of a malaria-free Nepal by 2025 and the country is currently in the pre-elimination phase [6].

At all endemic districts, there is a significant variation in malaria risk by regions, areas, and villages and they are largely dependent upon the local context. Thus, it is unlikely that a *one-size-fits-all* strategy will be appropriate for all endemic settings within a country [7]. This situation echoes Nepal’s current malaria epidemiology, where the risk of contracting malaria is highly variable from district to district and even between areas within the district [8]. In addition, within a district, the heterogeneity in malaria incidence and transmission is likely to complicate the interventions. Risk stratification will therefore be a key to tailoring the interventions within a country. Definition of priority areas for malaria control interventions should be based on the analysis of risk determinants related to the human host, parasite, and the vectors that together determine transmission intensity [7].

To date, Nepal has been implementing malaria control interventions targeting an entire district without selecting specific areas, despite that the risk varies within a district. An independent external assessment team commissioned by the World Health Organization (WHO) in 2010/2011 strongly recommended to update the existing stratification of malaria risk areas as early as possible [9]. Malaria risk mapping by micro-stratification up to the Village Development Committee (VDC) level was recommended in order to deploy appropriate and effective malaria control interventions to achieve a goal of malaria elimination by 2025. The main objective of this study was to estimate the risk of malaria at the VDC level in Nepal based on disease, vector, parasite, and geography.

## Materials and methods

### Study setting

Nepal has three main ecological zones (five ecological settings), mountain (middle- and high-range mountain), hill, and Terai (outer and inner Terai), running from west to east intersected by rivers flowing from north to south. In 2011, the population was estimated to be 26.6 million with an average family size of 4.9 persons. The annual population growth rate is 1.35%. An estimated > 1.9 million Nepali citizens, mostly male laborers, work outside of the country in India, the Middle East, and other countries, who upon return, contribute to the significant number of imported malaria cases. The four human malaria species are not evenly spread across the malaria-affected areas of Nepal. *P. vivax* is the most common species and predominates across all ecological settings, whereas *P. falciparum* predominates in the forest fringe, forest, foothills, and inner Terai. Urbanization continues to occur at a rapid pace, includes malaria endemic areas, and contributes to changing malaria transmission ecology.

### Study participants and study sites

The micro-stratification assessment was carried out in two phases: (a) in the first phase in 31 districts which reported more than 92% of malaria and 96% of *P. falciparum* of the total confirmed cases in the country and (b) in the second phase in the remaining 44 districts with few or no malaria cases (< 10% of total malaria cases).

### Study tools

Study tools were developed in consultation with experts. The VDC-based questionnaire had two parts. The first part included demographic, geo-ecological, meteorological, socioeconomic, and entomological information. The second part included malaria disease, diagnosis and treatment, classification, severity/death, drug resistance status, containment information, and vector control. Pre-testing of the developed documents was carried out in one Primary Health Center (PHC) of the *Kavre* district and was followed by necessary amendments, finalization, and endorsement.

### Data collection

**Fig. 1:**
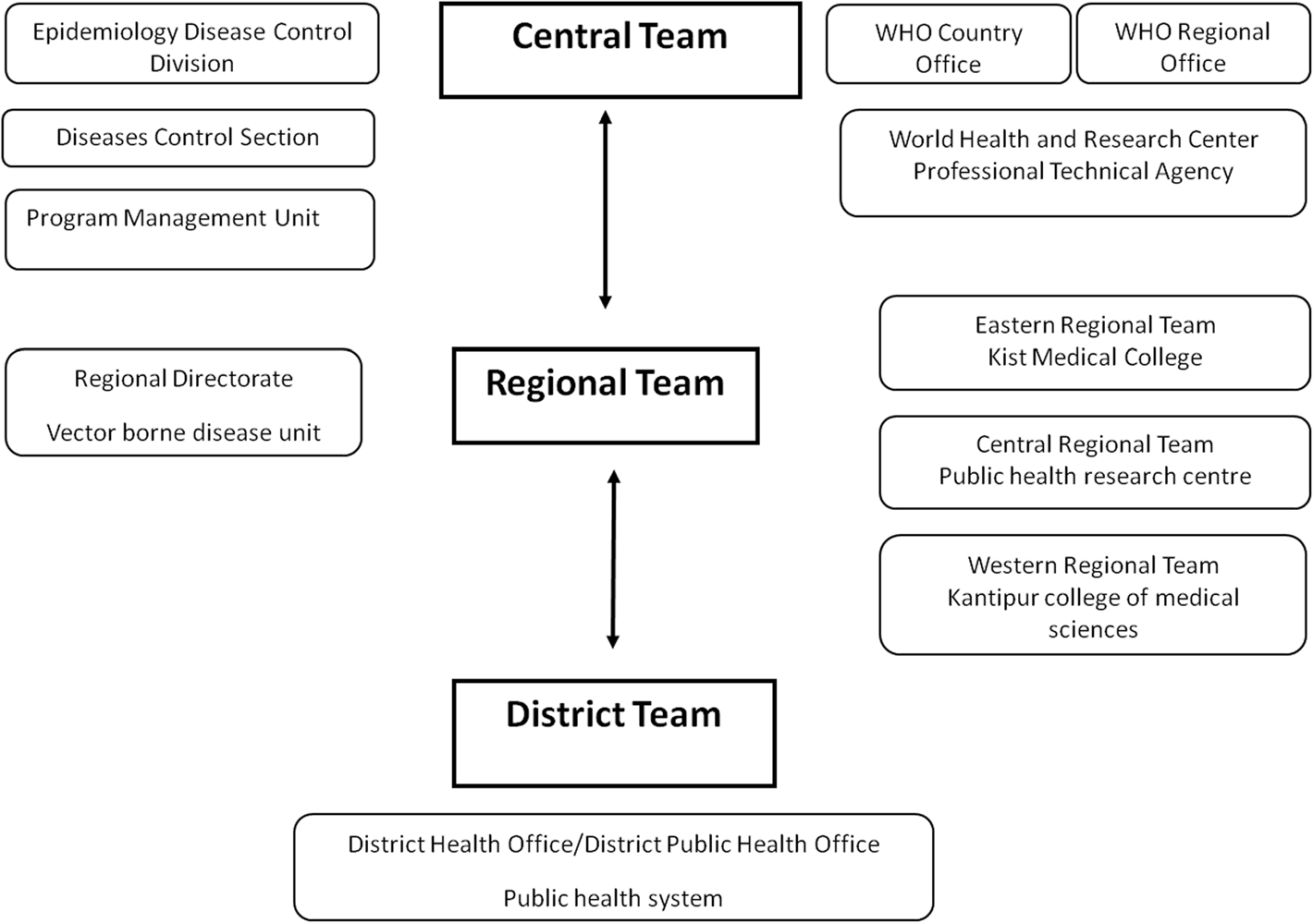
Composition and network of malaria micro-stratification teams

Micro-stratification teams were formed at three levels with clear reporting network at central, regional, and district levels to ensure credible data on basic malaria information by VDC/municipality (Fig. 1). The central team was composed of experts from the Epidemiology Diseases Control Division (EDCD), WHO country/regional offices, and other technical organizations. The central team collected essential data from the Central Bureau of Statistics, Department of Forestry, Department of Hydrology and Meteorology, Department of Local Development, and International Centre for Integrated Mountain Development (ICIMOD) and additional information from various organizations of national and international experts.

Five regional and 75 district teams were formed for data collection. Each regional team was led by an experienced entomologist, a data manager, and a lab technician and was supported by district teams (composed of district health officer/district public health officer (DHO/DPHO)). Both regional and district teams were apprised in advance on their roles and responsibilities in the assessment process of data that included collection, verification, validation, and compilation at the district and their collation at the center. A bi-directional line of communication mechanism was established between teams at all levels for efficient reporting and feedback.

Data was collected from April to September 2012. The regional teams were deployed at the districts for data collection and coordination with the district teams.

#### Data management

Collected data, including field reports by the regional teams, were validated using multiple mechanisms to ensure its credibility by the central team.

The accuracy of data was verified by the members of the central team and the EDCD through monitoring visits on selected study sites.

**Fig. 2:**
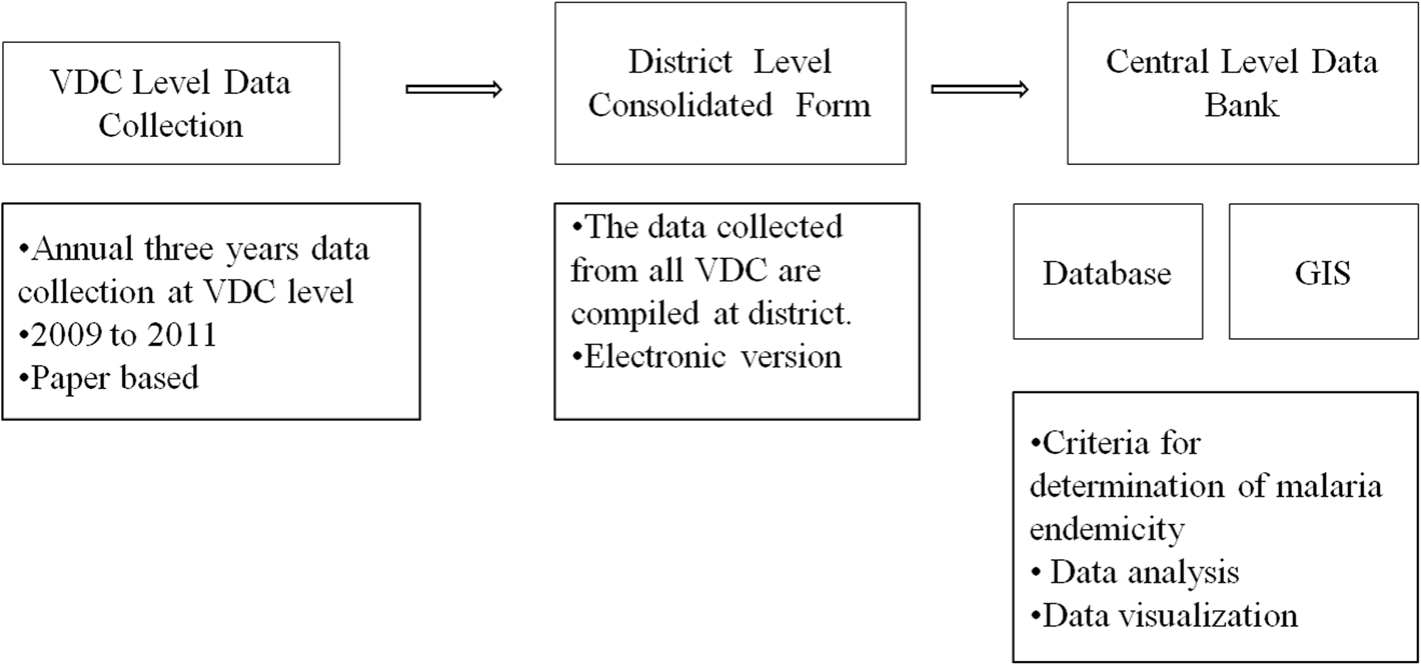
Basic malaria information flow from VDC to central database

A central database was established at EDCD consisting VDC level basic malaria information collected by the regional teams, population data, long-lasting insecticide-treated nets (LLINs) distribution data, drug resistance information from national experts, data regarding altitude (elevation) and land use from International Centre for Integrated Mountain Development (ICIMOD)*,* data from the Department of Meteorology and Hydrology, and data from the meta-analysis of existing malaria entomological reports generated by workshop (Fig. 2).

### Data analysis

#### Key determinants for risk analysis

VDC-wise basic malaria information was analyzed using exploratory statistics such as frequency and percentage in addition to geographical information system (GIS) to identify variables that contribute to malaria risk in Nepal. Three major variables (key determinants) were used: (i) disease burden average Annual Parasite Index (API-a), malaria cases per 1000 risk population; (ii) entomological risk at various ecological settings, ecology, and malaria vectors; and (iii) vulnerability and population movement.

#### Interpretation of data

Collected data was reviewed during a workshop on “Micro-stratification of malaria risk based on entomological findings in different ecological settings of Nepal.” The workshop was participated by senior malariologists, entomologists, and malaria control experts. All relevant documents were collected and interpreted. All available documents at EDCD and Vector Borne Disease Research and Training Center (VBDRTC), including papers published in national and international journals [10, 11], WHO reports [12], malaria entomological annual reports [13], personal collections, and other unpublished data, were scrutinized for vector characteristics and their bionomics in relation to malaria transmission. Each document was reviewed by individual experts and was followed by a focused group discussion to draw conclusion.

**Fig. 3:**
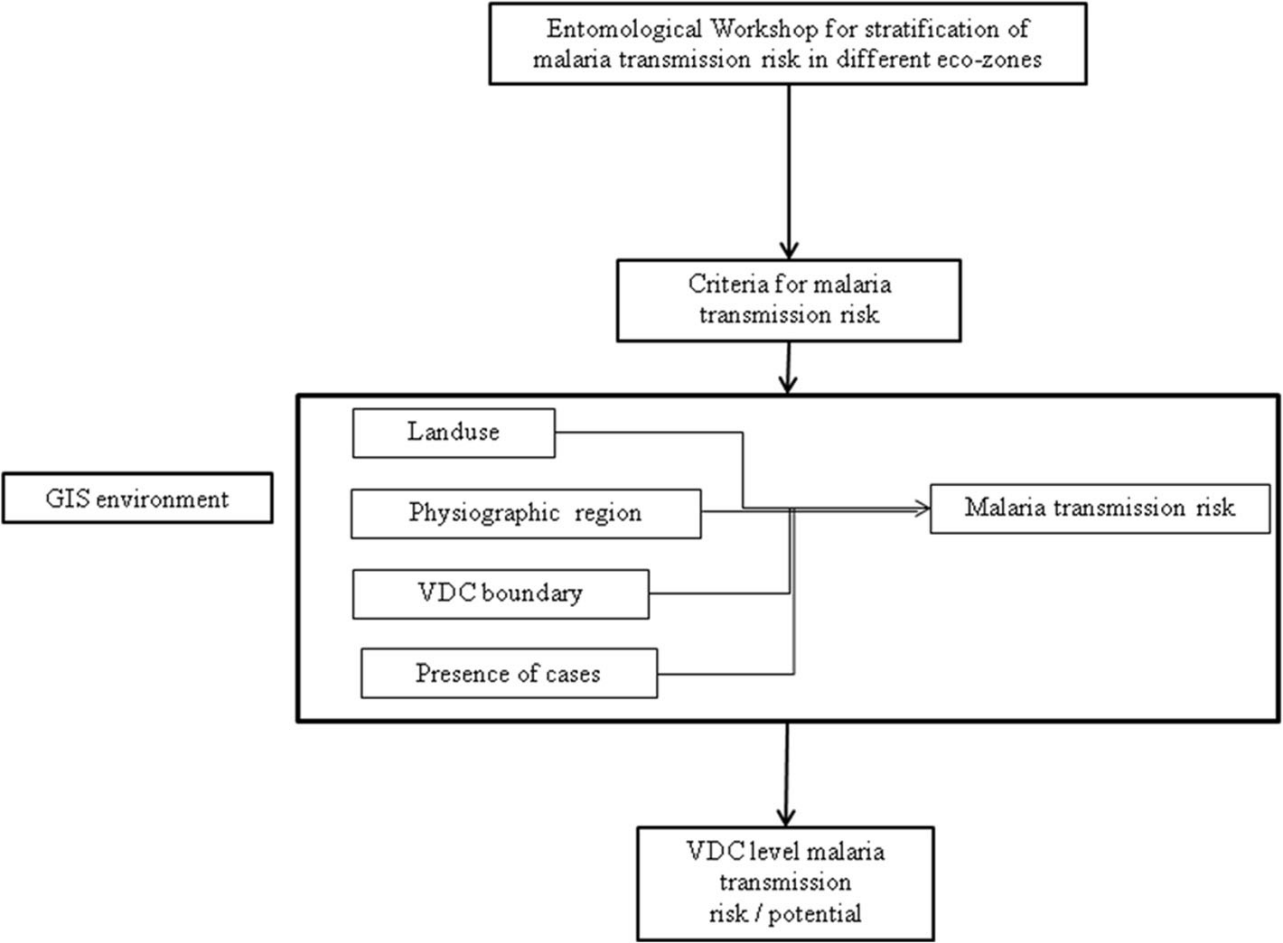
Flowchart showing steps to derive transmission risk by ecological setting

The workshop documented the characteristics of different malaria vectors and stratified the malaria risk according to the transmission potential of malaria vectors of Nepal in different ecological settings (Table 1; Fig. 3).

**Table 1:**
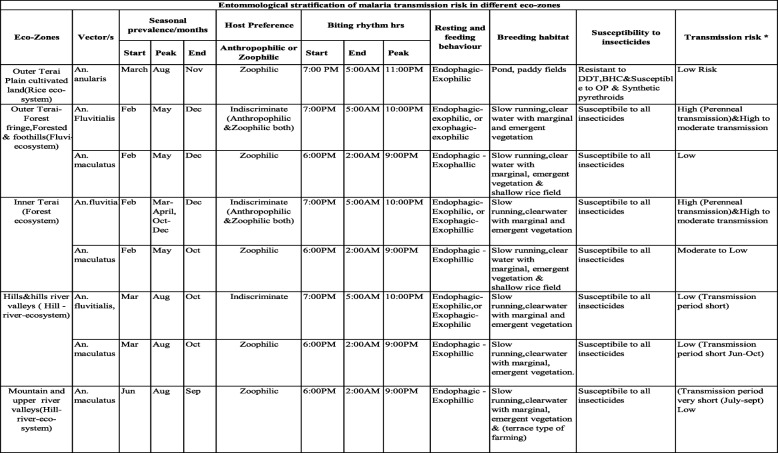
Entomological stratification of malaria transmission risk in different ecological settings of Nepal

*Risk criteria for malaria transmission is adapted as per recommendation made by Dr. G.B. White in 1982

#### Geographical information system analysis

To identify transmission risk at VDC level, overlay analysis was done in GIS environment using ArcGIS 10. Three GIS data layers, (i) land use derived from Thematic Mapper (TM), 2010; (ii) VDC boundary; and (iii) ecological zone, were overlaid. A VDC was considered in an ecological zone if a major part of the VDC fell in the zone. The same principle was applied for land use. A special consideration was given to the VDCs, which reported cases in all 3 years (2009 to 2011), showing the persistence of transmission, mostly in the inner Terai region, and was overlaid to further refine the malaria transmission risk by ecological settings.

#### Risk assessment and scoring method

Scoring methodology was selected to obtain cumulative risk after reviewing available analysis methods to ensure transparency, reproducibility, and comprehensibility of risk assessment. The methodology of assessment tool devised by the Center for Disease Control and Prevention (CDC), USA [14] was adopted to identify areas of malaria risk in Nepal. Each indicator establishes a weight (wt.) and then multiplies the weight by the response value to obtain a weighted value for each indicator. These weighted values were combined to construct the “overall risk score.” This methodology is implemented through different steps. Both qualitative and quantitative variables are converted to qualitative variables. A four-point Likert-type response is assigned to each variable. The assessment tool is presented in (Table 2).

**Table 2:** Scoring methodology for micro-stratification of malaria risk in Nepal

Level 1	Overall risk	Sum of level 2 X 100%		
Level 2 response* wt.	Indicators (wt.)	Disease burden (0.3)	Ecology (0.5)	Vulnerability (0.2)
Level 3	Variable response	API in 3 years	Transmission risk	Population movement
Response value	High (1.0)—H Mod (0.6)—M Low (0.1)—L No (0.0)—N	Average API ≥ 1.0—H Average API 0.01 to 0.99—M Average API is 0—L	Combination of geo-ecosystem and vector species (refer Table 1)	- Frequent movement to forests and development areas (high risk)—H - Visit to forest is infrequent but visit to high risk areas is frequent (moderate risk)—M - Movements to above areas are infrequent but endemic areas in Nepal or India is frequent (low risk)—L - Movement to non-endemic areas only (no risk)—N

*Level 2 response was categorized by a total of 100%

After the careful evaluation of four different scoring methods (applying different scores to different variables), it was decided on assigning (i) disease burden average API-a “0.3” wt., (ii) ecology-vector and transmission risk-a “0.5” wt., and (iii) vulnerability-population movement-a “0.2” wt. Utilizing this method, we provided much weight on ecology and transmission risk and it was considered epidemiologically credible because of the transmission risk potential that depends on ecology, vector, and transmission environment. Moreover, the main objective of micro-stratification was to delineate the areas according to the grade (level) of the risk of malaria transmission.

### Operational definition of risk

High-risk districts of Nepal are 25 districts of Terai region and 11 states of India. Vulnerability was calculated using a following categorical classification: movement to forests and development areas is frequent (high risk); visit to forest is infrequent but visit to high-risk areas is frequent (moderate risk), movements to above areas are infrequent but to endemic areas in Nepal or India are frequent (low risk), and movement to non-endemic areas only (no risk) (Table 2).

Operational definition of risk (*R*) was formulated to categorize and draw conclusions on malaria risk. Overall score ranged from 0 to 100, which was classified into four categories based on the operational definition.

*R* = (0.3 S1 + 0.5 S2 + 0.2 S3) × 100, where S1, S2, and S3 are the scores for API, entomology, and vulnerability, respectively, which were scored 0 for no risk, 0.1 for low risk, 0.6 for moderate risk, and 1 for high risk. The overall risk was classified into 4 classes: 0–20%, no risk; 20–50%, low risk; 50–80%, moderate risk; and 80–100%, high risk.

## Results

Malaria risk was stratified up to the VDC level on the basis of overall scores of the three major variables: disease burden, ecology/vector transmission, and vulnerability-population movement. VDCs were classified into four categories: no risk, low risk, moderate risk, and high risk (Table 3).

**Table 3:** Village Development Committees (VDCs) and risk population by development regions in Nepal

Region	Number of districts	High risk		Moderate risk		Low risk		No risk		Total VDC	
		VDC	Population^1^	VDC	Population^1^	VDC	Population^1^	VDC	Population^1^	VDC	Population^1^
Eastern	16	0	0	24	430,773	215	2,511,297	668	3,235,282	907	6,177,352
Central	19	10	258,959	77	927,902	353	2,821,865	778	5,675,767	1218	9,684,493
Western	16	7	115,595	27	372,822	191	1,881,278	652	2,661,812	877	5,031,507
Mid-western^2^	15	18	246,740	59	692,140	92	883,228	412	1,767,651	581	3,589,759
Far-western	9	19	364,342	14	237,055	148	1,281,067	208	799,408	389	2,681,872
Grand Total^2^	75	54	985,636	201	2,660,692	999	9,378,735	2718	14,139,920	3976	27,164,983

^1^VDC population is supplied by DPHO/DHO or else taken from census 2011, CBS

^2^VDC with missing population is not included in the analysis

*VDC* Village Development Committees, *DPHO/DHO* District Public Health Office/District Health Office, *CBS* Central Bureau of Statistics

### Disease burden

**Fig. 4:**
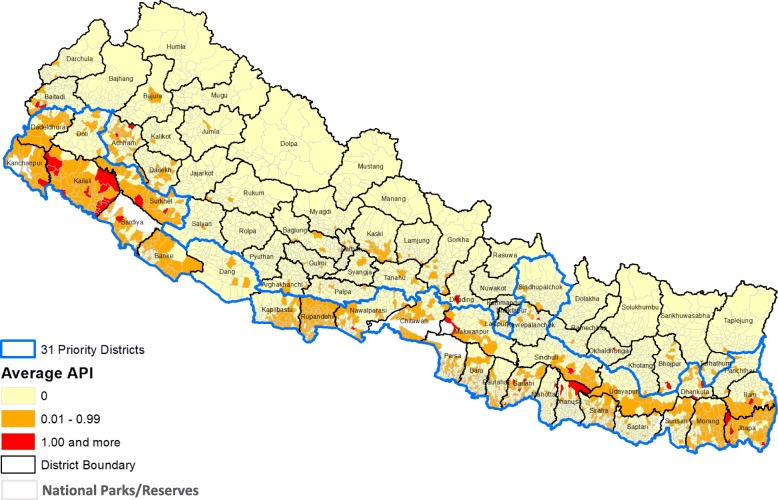
Average annual API from 2009 to 2011 (disease burden)

Of the total VDCs (*n* = 3976; 4 excluded for missing values), 44 (1.10%) reporting API-a ≥ 1, 752 (18.91%) reporting API-a = 0.01–0.99, and 3176 (79.88%) reporting API-a = 0 (no malaria cases) were respectively classified as high-, moderate-, and low-burden VDCs based on a 3-year analysis (2009 to 2011) (Fig. 4). VDCs with high, moderate, and low burden received a response value of 1.0, 0.6, and 0.1, respectively.

### Entomological risk/ecology

**Fig. 5:**
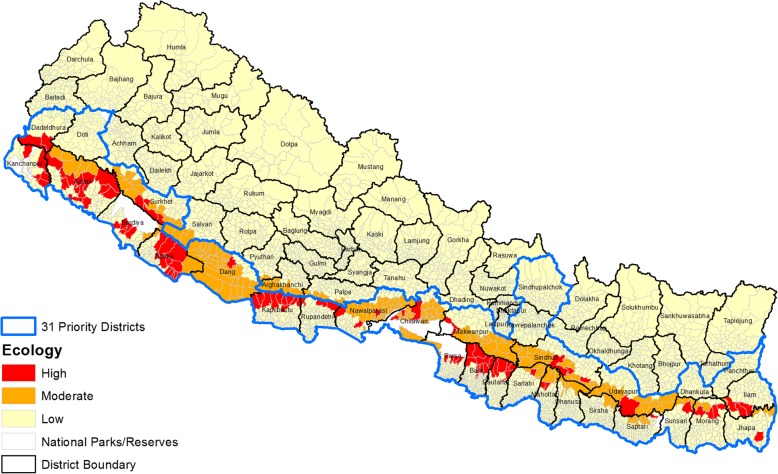
Entomological risk of malaria transmission (Ecology)

Entomological risk was derived based on the historical evidences (Table 1) using the combination of ecological zones (five ecological settings), land-use pattern, and trend of malaria cases analyzed in GIS environment. Altogether, 97 (2.44%), 206 (5.18%), and 3665 (92.18%) VDCs were respectively classified as high, moderate, and low transmission potential. Upon further refinement based on land use, the outer Terai showed two distinct high and low transmission potential areas that included forest and cultivated areas (Fig. 5). Inner Terai showed high and moderate transmission potential, leaving the rest of the three ecological settings under low transmission potential. Output refinement in the inner Terai yielded two categories: high (VDCs having cases in all 3 years) and moderate transmission potential (cases in any of the 3 years). Interestingly, this refinement changed 23 (1.17%) inner Terai VDCs from moderate to high transmission potential.

### Vulnerability

**Fig. 6:**
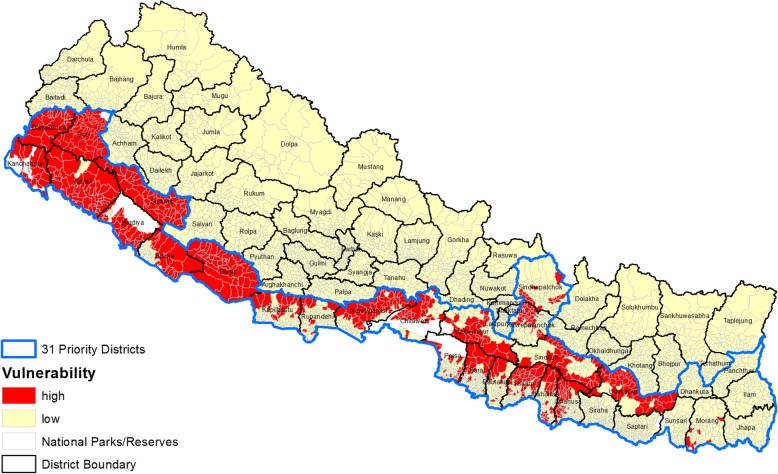
Vulnerability due to population movement

There were 686 (17.25%) and 3290 (82.75%) VDCs under high and low vulnerability, respectively, but none of the VDCs were moderate and non-vulnerable (Fig. 6).

### Overall risk

**Fig. 7:**
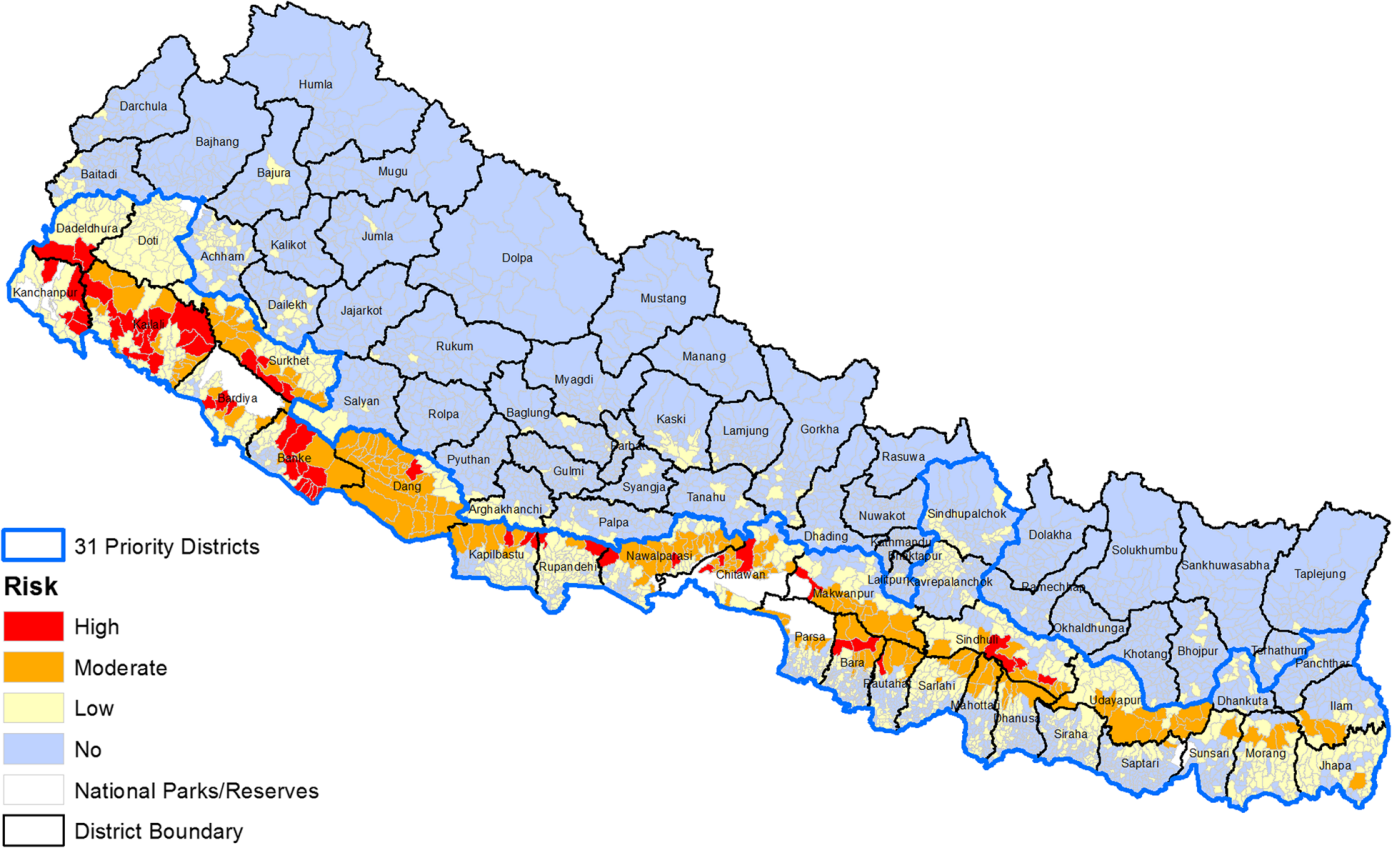
Village Development Committee (VDC) level malaria risk in Nepal

Analyzing the overall risk based on scoring (disease burden, 0.3 wt.; ecology, 0.5 wt.; and vulnerability, 0.2 wt.) of the total VDCs (*n* = 3976), 54 (1.36%), 201 (5.06%), 999 (25.13%), and 2718 (68.36%) VDCs were identified as high-, moderate-, low-, and no-risk categories for malaria, respectively (Fig. 7).

Based on the population statistics, 3.62% (985,636/27,164,983), 9.79% (2,660,692/27164983), 34.52% (9,378,735/27164983), and 52.05% (14,139,920/27164983) of the country’s total population live in high-, moderate-, low-, and no-risk VDCs, respectively. Regional distribution showed that the majority of the high-risk VDCs (1918 VDCs) were identified in the Far- and Mid-western regions followed by Central and Western regions (107 VDCs) with no high-risk VDC in the Eastern region. Similarly, 77, 59, 27, 24, and 14 VDCs of the Central, Mid-western, Western, Eastern, and Far-western regions, respectively, were found to be under moderate malaria risk. Of the low-risk VDCs, 353, 215, 191, 148, and 92 were respectively from Central, Eastern, Western, Far-western, and Mid-western regions (Table 3).

## Discussion

With the significant reduction of malaria cases in recent years, the malaria control program in Nepal has set up the vision of “a malaria-free Nepal by 2025” and the country is currently in the pre-elimination phase (Table 4) [5, 15]. Malaria risk mapping by micro-stratification up to the VDC level is critical in order to deploy appropriate and tailored malaria control interventions to achieve the targeted goal of elimination. Micro-stratification helps to highlight the distribution and potential impact of multiple disease interventions [16]. This approach should be used more widely over time and space and at different geographical scales to better monitor and understand the impact of single and multiple interventions and to assess progress towards the elimination of different diseases [17]. Although not commonly practiced in malaria control programs, such an approach is necessary for national planning purposes as well as increasing the cost effectiveness and coordination of malaria elimination programs where different strategies are deployed.

**Table 4:** Recommended interventions per stratum for Nepal malaria program

Interventions	High risk	Moderate risk	Low risk	No risk
LLINs	First priority; limited to wards with indigenous cases and adjacent wards within 2–3 km	Second priority; limited to wards with indigenous cases adjacent wards within 2–3 kms	Third priority; limited to households with confirm cases only to prevent transmission	NA
IRS	Focal; 1–2 cycles depending on the duration of transmission and residual efficacy of insecticide	Yes; to contain outbreak	Yes, to contain outbreak	NA
Larval control	As appropriate	As appropriate	As appropriate	NA
EDPT	Yes	Yes	Yes	Yes
Case investigation	Yes	Yes	Yes	Yes
Foci investigation	Yes, second priority	Yes, first priority	No, except when indigenous case is reported	NA
BCC	Yes	Yes	Yes	Yes

*LLINs* long-lasting insecticide-treated nets, *IRS* indoor residual spraying, *EDPT* early diagnosis and prompt treatment, *BCC* behavioral change communication, *km* kilometers, *NA* not applicable

The application of micro-stratification to control diseases has been increasingly adopted in recent years. For example, micro-stratification has been utilized in malaria control and elimination program in Timor Leste [18]. In Philippines, micro-stratification has been applied to control and eliminate vector-borne diseases such as dengue and malaria [19].

The malaria risk stratification in Nepal considers several key determinants of malaria transmission, for example, disease burden (API–malaria cases per 1000 risk population) in the last 3 years, ecology that determines the presence of the vectors, relative efficiency of the vectors in malaria transmission, duration of transmission in ecological zones, and vulnerability in terms of population movement. The key determinants (termed as major variables) are given weights to stratify the malaria risk. This method has been used to control lymphatic filariasis in the Democratic Republic of Congo [20] and Nigeria [16]. Overall, the stratification of malaria risk areas is robust enough to be used for the planning and implementation of key interventions [21]. This information is a prerequisite for effective planning and will help to appropriate the elimination strategy to ensure targeted coverage to be cost effective with maximum impact [11, 18].

In the context of Nepal, there is a large variation in topography and ecology in many of the VDCs. VDC, as a unit of the study, may have generalized these variations. Stratification by a VDC comprises several wards and villages. However, in Nepal, there was no systematic malaria risk assessment and the previous risk assessments were limited to district level [22]. The current micro-stratification provides the insight of malaria risk at VDC level. This will help the Malaria Elimination Program to target interventions at the VDC level, thereby ensuring the best utilization of available resources to substantially narrowed-down target areas [16]. In neighboring country Bangladesh, mapping the stability of malaria hotspots has been under operation to achieve the goal of informing intervention planning for malaria elimination [23].

In many areas of Nepal, there are other diseases which are co-endemic with malaria. Therefore, micro-stratification can also be used as an integrated approach to facilitate multiple disease control and elimination such as lymphatic filariasis. In African settings, such an approach has been found greatly effective in planning and implementing the programs against co-endemic conditions such as loiasis and filariasis [20]. Current micro-stratification of malaria work builds on the recent study carried out in the Democratic Republic of Congo [20], which used the new overlap mapping approach to collate and map all available country data on *Wuchereria bancrofti*, examine the extent of *Loa loa* co-endemicity, and determine the risk and benefits of different intervention strategies.

### Implications for malaria control and elimination

Malaria control and elimination strategies are being accelerated in Southeast Asia and the Greater Mekong region and have mainly focused to halt the spread of artemisinin resistance using multiple control measures such as mass drug administration for malaria hotspots, strengthening village malaria workers, and deployment of LLINs with community engagement strategies wherein community members are trained and devolved with the responsibilities in the malaria risk areas which can be enhanced by malaria micro-stratification study [24–32]. Even though a recent study from Nepal has not yet shown the artemisinin resistance against *P. falciparum* in Nepal, a continuous monitoring for resistance markers was recommended to be critical [15]. In addition, studies from Nepal have shown *P. vivax* as a dominating species for the last 50 years [5]. In recent years, malaria vectors have been isolated in the hilly regions of Nepal, which previously were devoid of vectors for malaria [22, 33]. The control and elimination of malaria, therefore, needs to target species-specific interventions integrating the current elimination program [34]. For the complete elimination of malaria (including *P. vivax*), a radical therapy using primaquine is essential. Nevertheless, administration of radical therapy using primaquine requires G6PD deficiency testing [35–37]. Although the current study focused on *P. falciparum*, future micro-stratification study can benefit by expanding micro-stratification by species as well. In order to target the malaria risk areas (low-, moderate-, and high-risk areas for malaria) identified by the current micro-stratification study, community engagement strategies might be beneficial to intensify malaria control activities [24, 25, 31, 38].

### Limitations

The entomological information integrated into this study is not up to date. There is no current study conducted in entomology. Over the years, there is a massive scale of ecological changes including high usage of insecticides that can yield changes in vector bionomics. The analysis is based on historical evidences only. There are no baseline entomological studies of the Himalayan mountain districts, and it is presumed that there are no malaria vectors present in the region due to the climatological factors and based on the history of no evidence of malaria transmission.

A large number of missed clinical cases may have been underestimated in this study to classify the areas with malaria risk. This study did not include cases from private facilities.

## Conclusion

The current micro-stratification estimates the population at risk of malaria has decreased to 13.02 million from 20.35 million over the past 5 years. This study has re-defined the malaria risk and re-mapped it more systematically. Findings from this study can aid in utilizing the available resources to substantially narrowed-down target areas. In future, micro-stratification can be employed to further identify the risk areas into smaller units within the VDCs such as wards and villages.

## Abbreviations

API: Annual Parasite Index
BCC: Behavioral change communication
CBS: Central Bureau of Statistics
CDC: Center for Disease Control and Prevention
DHO: District Health Office
DPH: District Public Health Office
EDCD: Epidemiology and Disease Control Division
EDPT: Early diagnosis and prompt treatment
GIS: Geographical information system
GPS: Global Positioning System
HMIS: Health Management Information System
ICIMOD: International Centre for Integrated Mountain Development
IRS: Indoor residual spraying
LLINs: Long-lasting insecticide-treated Nets
NMP: National Malaria Program
RDT: Rapid diagnostic test
SOPs: Standard operating procedures
VBDRTC: Vector Borne Disease Research and Training Center
VDC: Village Development Committee
WHO: World Health Organization
